# Negative plant-soil feedbacks disproportionally affect dominant plants, facilitating coexistence in plant communities

**DOI:** 10.1038/s44185-023-00032-4

**Published:** 2023-12-21

**Authors:** Elias P. Goossens, Vanessa Minden, Flor Van Poucke, Harry Olde Venterink

**Affiliations:** Department of Biology, Vrije Universiteit Brussel, Pleinlaan 2, 1050 Brussels, Belgium

**Keywords:** Ecology, Biodiversity, Community ecology, Ecosystem ecology, Grassland ecology, Invasive species

## Abstract

Plant-soil feedbacks (PSFs) are suggested to be major drivers of plant species coexistence and exotic invasions in natural plant communities, where species with more positive PSFs are thought to be more abundant in communities. Most evidence for this comes from mesocosm experiments with single species, but whether the results are transposable to diverse plant communities is mostly not verified and remains debated. We performed a combined monoculture and community experiment to test whether PSFs in monocultures predict PSFs in communities, and to infer the role of PSFs in invasive plant success. We found that (1) PSFs from monocultures were poor predictors for PSFs in plant communities, (2) competitive strength of invasive species did not consistently depend on PSF, and (3) dominant species experienced a significantly stronger negative PSFs than non-dominant species when grown in community. Hence, PSFs of plant species in monocultures seem less predictive for their abundance in plant communities or for invasibility than previously assumed. Nevertheless, PSF—and particularly negative PSF—seems indeed a major driver of plant species coexistence, with a strong species-specific pathogenic effect on dominant plants facilitating the persistence of rare species.

## Introduction

Plant-soil feedbacks (PSF) have been suggested to be an important regulating mechanism for plant species dynamics in grassland communities^1,2^. The accumulation of mutualistic mycorrhizal fungi in the rhizosphere induces positive PSFs around conspecific plants^3^, while soil-borne pathogens can induce negative PSFs by hampering their growth or that of their offspring^4–6^. These species-specific pathogens are one of the major drivers of species richness-productivity relationships^7,8^. PSF has also been proposed to influence community structure by benefitting the establishment of invasive species through positive feedbacks^9,10^ and species coexistence through negative or positive feedbacks^11–13^. The enemy release hypothesis (ERH)^14^ postulates that non-native plants can become invasive because species-specific pathogens are absent in the introduced range^15,16^. It has indeed been shown that invasive plants often encounter more positive or less negative PSFs than natives^17,18^, though some studies showed the opposite^19–21^.

In what manner PSF influences plant species dominance is debated^22^. Some studies showed that dominant plant species have net positive PSFs or weaker negative PSFs than co-occurring rare species^17,23,24^, whereas other studies found that PSF has negative effects on most plants^12^, with dominant plant species accumulating more species-specific pathogens^3^, maintaining plant diversity through negative frequency dependency^2,12,13^. Although it has been theoretically predicted that negative PSFs can promote plant species coexistence^25–29^, supporting experimental evidence is lacking.

Most experimental setups so far measured PSF in monoculture mesocosm experiments, assigning fixed PSF values to plant species^17,30–32^, though some works have suggested that interspecific competition in communities between plants may alter individual PSFs^33–36^. For example, more recent experiments have shown that PSFs from monocultures are not correlated to these found in the field^37,38^. Hence, there is a need for community experiments^12,39,40^, where the interaction between competition and PSF can be measured. Some earlier experiments indeed did investigate PSF in artificial communities^21,34,41–43^, though these only included pairs of two to three species or did not focus on the effect of communities on PSF.

The aim of this research was to investigate if monoculture mesocosm experiments are good predictors for PSFs in plant communities. We therefore combined monoculture and community PSF experiments for 10 co-occurring native grassland species and three invasive species. The communities consisted of these 10 native species with or without one of the three invasive species. We either used a full microbial inoculum (PSF_tot_) or an extracted pathogen/saprobe filtrate (PSF_path_) from the soil of the conditioning phase of the experiments to determine PSF for each species in both monoculture and community in the response phase (see Extended Data Fig. 1). A sterilized inoculum was used as control. If PSFs of species in monocultures are good predictors for PSF in communities, then this species-specific PSF might indeed influence plant dominance^17^. Species with more positive PSFs in monoculture will thrive more in unsterilized communities than in sterilized ones, leading to increased size or dominance, unlike species with more negative PSFs in monoculture. On the other hand, if monoculture experiments appear to be poor predictors of PSFs in community, then what factors contribute to PSFs in community that disrupt the correlation with monoculture PSFs? We hypothesize that in this alternative case, PSF in communities is affected by plant dominance^44^, where large, dominant species will accumulate more pathogens and thus receive a more negative PSF^3^. The PSF a species receives then depends on that species’ dominance within the community^26,27,29^. Lastly, we tested whether the enemy release hypothesis applied to the three invasive species of our experiment (*Solidago gigantea*, *Avena sterilis* and *Lupinus polyphyllus*). We hypothesized that these species are stronger competitors and represent higher proportions of the community biomass in unsterilized communities than in sterilized communities.

## Results and discussion

**Fig. 1:**
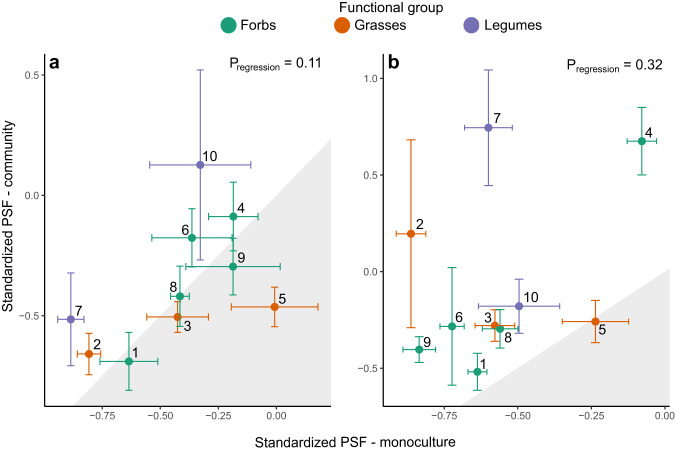
PSFs for all native species grown in community (without invasives) in function of the PSFs of these species when grown alone, calculated for their total ground biomass. **a** Shows results for standardized PSF_tot_ from both mutualists and pathogens. **b** Shows results for standardized PSF_path_ from the pathogen fraction alone. Grey areas show when PSF is more positive when grown alone than in community, for white areas PSF is more positive in communities. Significance of regression was evaluated using a linear model (see ‘Methods’). Error bars indicate standard errors, *n* = 5 for calculation PSF in community per species per soil treatment, only communities without addition of invasives were used; *n* = 6 for monocultures. Species numberings: 1. *Achillea millefolium*, 2. *Agrostis capillaris*, 3. *Anthoxanthum odoratum*, 4. *Centaurea jacea*, 5. *Holcus lanatus*, 6. *Leucanthemum vulgare*, 7. *Lotus corniculatus*, 8. *Plantago lanceolata*, 9. *Rumex acetosa*, 10. *Trifolium pratense*.

For most species both PSF_tot_ and PSF_path_ were negative regardless of being grown in monoculture or community (Fig. 1, Extended Data Figs. 2–4). However, for nine out of ten species PSF_path_ was less negative in community than in monoculture, showing that the impeding effect of pathogens on plant growth was reduced when grown in community (Fig. 1b). These results are in line with earlier meta-analyses, where monocultures grown in the greenhouse showed more negative feedbacks than field experiments^38,40^. This could result from pathogens often being species-specific^3,17^: while in monocultures the entire pathogen fraction will be represented by the same species-specific pathogens, in more biodiverse communities pathogens specific for each plant species will occur in lower proportions. Since most mutualistic arbuscular mycorrhizal fungi are generalists^45^ this negative effect of higher plant species diversity is probably less relevant for positive feedbacks. Due to this asymmetry in specificity, PSF might become less negative with rising biodiversity of the plant community.

**Fig. 2:**
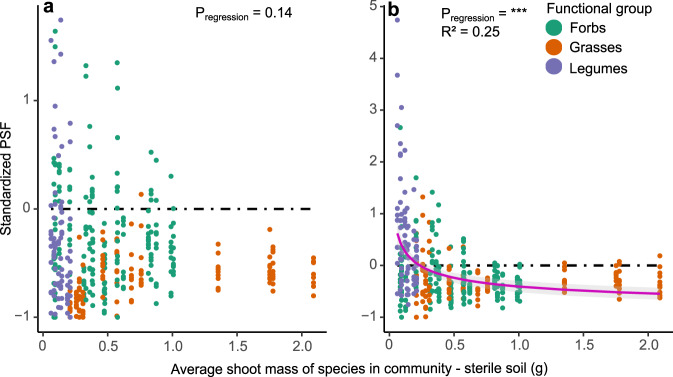
PSFs for all native individuals grown in community in function of their averaged biomass when grown in sterile soil, calculated for their above-ground biomass. **a** Shows results for standardized PSF_tot_ from both mutualists and pathogens. **b** Shows results for standardized PSF_path_ from the pathogen fraction alone. Significance of regression was evaluated using a mixed linear model (see ‘Methods’). R² indicated on the graph is the marginal R², conditional R² = 0.31. Trendline was made with the function nls, y = 0.59/x^0.36^ – 1, all parameters highly significant, CI = 99.5%.

We found that PSFs measured in monocultures were not correlated to these in communities (Fig. 1a, b, resp. R² = 0.29 & 0.12, Ext. Data Fig. 2a, b, resp. R² = 0.06 & 0.03). Our results confirm that PSF measured in monoculture experiments are thus not representative for community, and consequently neither for field conditions^37,38^. Since most earlier works on PSF mechanisms concentrated on monoculture experiments, we see a clear need for individual PSFs from different plant species measured in a community setting, in order to fully explain how PSF and community structure influence one another. As far as we know, only one earlier study was able to show a significant correlation between PSF in monocultures and plant abundance in the field^17^.

We here show that larger, more dominant species (in terms of biomass) in our experimental community were exposed to significantly more negative feedbacks than smaller species (Fig. 2a, b for above-ground biomass, Extended Data Figs. 5, 6 for below-ground biomass and total biomass, for both PSF_tot_ and PSF_path_, Table 1). We attribute this dominance-driven negative PSF to pathogen fractioning, where pathogens specific for low-abundant or smaller species represent low proportions of the soil community, in contrast to pathogens specific for more dominant or larger plant species. Since this mechanism is not applicable in monocultures, PSF was here not dependent on species biomass (Extended Data Fig. 7a, b, Table 1). Some of the smaller species in the community, such as *Lotus corniculatus* and *Centaurea jacea* even showed a significantly positive PSF_path_ in community whereas they had a negative or insignificant PSF_path_ in monoculture (Fig. 1 and Extended Data Figs. 2–4). This seems to be an indirect effect of the larger species being stronger affected in communities by negative PSF, enabling a better performance of the smaller or rare species in the community in the presence of soil microbes through a reduced competition from these larger species. The PSFs in community, especially for smaller, less competitive plants, are thus a combination of the direct feedback induced by its own species-specific soil biota, and of the indirect effect from altered competition with other plant species and their PSFs. Though PSF_path_ can never be positive for a certain species in monoculture (since mutualists are absent), we here show that it can become positive for these small species in communities, demonstrating that the positive effects from the reduced competition can overwhelm the negative effect of their own species-specific pathogens. PSF can thus be a strong driver of community composition and species coexistence, in contrast to conclusions of some earlier works which found that PSF in community seemed to be of minor importance due to their effects being overwhelmed by other interactions^37,40,43^. Our results support that negative PSFs in community can promote plant coexistence and thus biodiversity through negative frequency dependency^12,27^. Just as PSF alters with e.g. water availability^46^ or nutrient availability^47,48^—it also changes depending on the community in which the plant occurs and its dominance within that community. This negative frequency dependency seems most significant when calculated for root biomass (Extended Data Fig. 5a, b, Table 1). This might be explained by soil pathogens being associated with the rhizosphere of plants, making root biomass the main determinant for pathogen load, thus also PSF. The effect is also significant when calculated for above-ground and total biomass, though only for PSF_path_ (Fig. 2b, Extended Data Fig. 6b, Table 1). The negative frequency dependency also seems to be present for the PSF_tot_ (Fig. 2a), though not significant (*P* = 0.14). The presence of mutualists may obscure the directional effect of pathogens. This effect could perhaps be explained by different plant species benefitting to varying degrees of mutualistic mycorrhizal fungi^49^. Plant communities more dependent on mutualistic mycorrhizae, or where mutualists are more abundant, might show a reduced negative frequency dependency.

**Table 1:** (Mixed) linear model results showing how PSF_tot_ and PSF_path_ for each species are impacted by its biomass and functional group.

		Species biomass		Functional group		Interaction		Random factors	R²
		χ^2^	*P*	χ^2^	*P*	χ^2^	*P*	Variation expl. (%)	Marg.–cond.
Above GB	PSF_tot_	2.2	0.14	59.4	***	–	–	1.4	0.13–0.14
	PSF_path_	26.9	***	24.5	***	25.1	***	1.3	0.30–0.31
Below GB	PSF_tot_	20.9	***	4.2	0.12	–	–	0.9	0.09–0.10
	PSF_path_	43.9	***	5.7	0.057	–	–	0.6	0.19–0.19
Total B	PSF_tot_	0.0026	0.96	25.3	***	–	–	2.6	0.11–0.13
	PSF_path_	14.6	***	16.5	***	25.5	***	2.0	0.38–0.40
Monoculture (Above GB)	PSF_tot_	0.3^a^	0.58	6.3^a^	**	–	–	–	–
	PSF_path_	2.0^a^	0.17	0.008^a^	0.99	–	–	–	–
ΔPSF (Comm.–mono.)	PSF_tot_	10.1	**	–	–			83	0.19–0.87
	PSF_path_	6.8	**	–	–			0	0.43–0.43

The first column shows the effect of species biomass in sterile soil on the PSF it receives when grown with soil treatment. The second column shows if this PSF depends on the species’ functional group. The third column shows if the interaction term between species biomass and functional group was significant. The fourth column shows the variation explained of all random factors (see ‘Methods’). The last column shows R² values (marginal and conditional) of the mixed linear models. The first three rows show results for the community experiment. The fourth row shows results for the monoculture experiment. The last row shows results for the difference in PSF between community and monoculture. *P*-values: *** < 0.001 < ** < 0.01 < * <0.05 < . < 0.1 <–.

*Above GB* Above-ground biomass, *Below GB* Below-ground biomass, *Total B* Total biomass.

^a^F values are given for linear regressions made for monoculture experiment.

Furthermore, grasses showed significantly more negative PSF than legumes or non-leguminous forbs in communities (Fig. 2a, b and Extended Data Figs. 6a, b, *P* < 0.001 for both PSF_tot_ and PSF_path_, Table 1), though this effect was not present in monocultures (Extended Data Fig. 7a, b). These results are in line with the meta-analysis by Kulmatiski et al.^12^, who attributed this to high root to shoot ratios and related to that a higher vulnerability to pathogens. Our results support these results that grasses seem to accumulate more pathogens rather than that they benefit less from mutualists, as this trend was significant for both PSF_tot_ as for PSF_path_. Other than simply accumulating more pathogens, grass-specific pathogens might be less species-specific, where pathogens can affect different closely related plant species^24,50^, though we are not aware of any studies showing this specifically for grasses. Lastly, though PSF is significantly negatively impacted by species size in community for all functional groups, this negative dependency on size seems to be stronger for legumes (significant interaction term between species biomass and functional group, see Table 1).

**Fig. 3:**
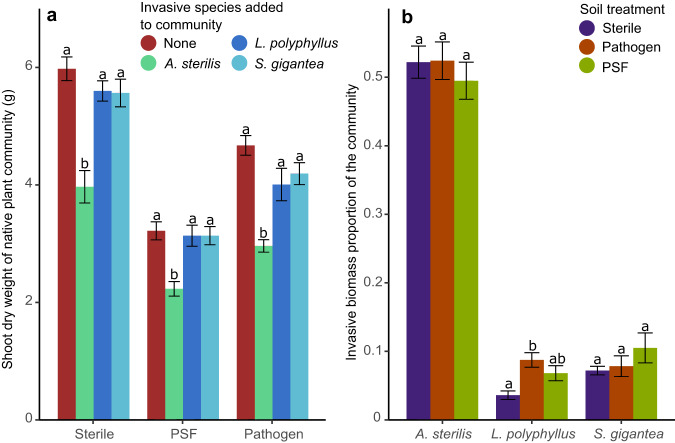
Impact different plant soil feedback treatments and/or presence of an invasive plant species on community biomass of native plants. **a** Above-ground community biomass of 10 native plant species in response to soil treatments and presence/absence of one invasive species. Sterile = sterilized soil treatment, PSF = unsterilized soil treatment, Pathogen = sterilized soil treatment + addition of pathogen/saprobe filtrate. **b** Biomass of the invasive plants (% of total above-ground community biomass) in relation to soil treatments. Letters indicate significant differences depending on **a** the presence of an invasive species or **b** soil treatment, calculated with Tukey’s HSD test, *α* = 0.05, *n* = 10. Error bars indicate standard error. *A. sterilis* *Avena sterilis*, *L. polyphyllus* *Lupinus polyphyllus*, *S. gigantea* *Solidago gigantea*.

Assuming that the enemy release hypothesis applies to the three investigated invasive species of our study, we expected these species to be less negatively impacted by PSF than native species, making them stronger competitors^17,18^. However, the competition effect of invasive species on native species was independent of soil treatment in our study (interaction term not significant in Table 2, Fig. 3a, Extended Data Fig. 8a). Along with this, two out of three invasive species did not significantly represent a higher proportion of the total community biomass depending on soil treatment (Fig. 3b and Extended Data Fig. 8b; Table 2). The only invasive species that did represent a significant higher proportion of the community when PSF was present was *Lupinus polyphyllus*, which was also the only species to receive a positive PSF_tot_ in monoculture (Extended Data Fig. 3). Hence, our results do not generally support the ERH, and are in line with other studies emphasizing that invasion processes are complex, and cannot fully be explained by the ERH alone but also depends on other factors^19^.

**Table 2:** ANOVA results showing how the growth of natives (two-way ANOVA) and invasives (one-way ANOVA) is impacted by soil treatment and competition.

		Soil treatment		Invasive species		Soil treatment × Invasive species	
		F	*P*	F	*P*	F	*P*
Natives	AGB	153.4	***	39.3	***	1.6	–
	TB	54.6	***	2.3	.	0.2	–
*L. polyphyllus*	AGB	7.5	**				
	TB	13.4	**				
*A. sterilis*	AGB	0.4	–				
	TB	0.2	–				
*S. gigantea*	AGB	1.3	–				
	TB	1.8	–				

The first column shows the effect of the different soil treatments on biomass of all natives combined and on the three invasive species. The second column shows if the addition of an invasive species to the community has significant effects on native biomass. Four different addition possibilities: (i) no invasive species present, (ii) invasive present, *Avena sterilis*, placed in the middle of the community, (iii) invasive present, *Lupinus polyphyllus*, (iv) invasive present, *Solidago gigantea*. The last column shows if the interaction of soil treatment and the addition of an invasive species has a significant effect on native biomass. *P*-values: *** <0.001 < ** <0.01 < * <0.05 < . < 0.1 < –.

*AGB* Above-ground biomass, *TB* Total biomass.

As discussed above, PSF seems to become less negative with increasing biodiversity of the plant community due to species-specific pathogens representing lower proportions of the total pathogen fraction. The validity of the ERH diminishes as the biodiversity of the invaded community increases, causing invasive species to lose their competitive edge. Invasiveness due to enemy release might thus mostly apply in communities of low biodiversity. It has already been shown theoretically and experimentally that more biodiverse communities are more resistant to invasion than communities of low diversity^51–53^. In these studies, low invasibility was predicted to result from the presence of species of many different functional guilds^52^ or the low levels of resources^51^ that co-occur with diverse communities. The reduction in negative PSF for natives and accompanying larger competition experienced by invaders due to the higher native biomass production might be an additional explanation, as it has been shown that biodiversity increases productivity^54^ due to a reduced species-specific pathogen load^7,8^. As plant-pathogen relationships are often species-specific, we predict that the biodiversity effect on plant invasions through pathogens does not saturate with increasing biodiversity due to functional similarity of species^55^, but is more in line with the singular hypothesis of biodiversity where each plant-pathogen interaction is unique^56,57^. Rather than actively driving the decrease in diversity and nutrient enrichment^58–61^, the invasion of some alien species may be a consequence of these disturbances^43,62,63^, e.g. in communities that lost diversity through nutrient enrichment, where these invasive species could again benefit from the ERH.

Our results do confirm that on a community scale PSF has a significant net negative effect on biomass production^12^ (Fig. 3a for above-ground biomass, Extended Data Fig. 8a for total biomass; Table 2). Besides this, the addition of an invasive plant species may also significantly reduce total native biomass (Fig. 3a and Table 2), though this was only significant for one out of three species in our experiment (*A. sterilis*). The other two invasives also reduced native biomass, though not significantly.

**Fig. 4:**
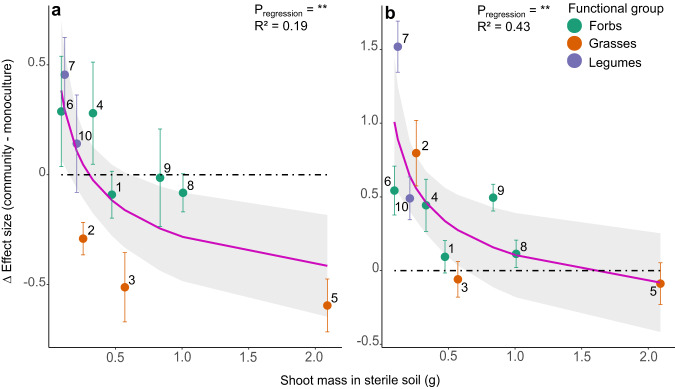
Difference in PSF between community grown and monoculture grown plants (ΔPSF) in function of their averaged biomass when grown in sterile soil in community, calculated for their above-ground biomass. **a** Shows results for ΔPSF_tot_ from both mutualists and pathogens. R^2^ indicated on the graph is the marginal R², conditional R^2^ = 0.87. Trendline was made with the function nls, y = 0.72/x^0.28^ – 1, all parameters significant, CI = 95%. **b** Shows results for ΔPSF_path_ from the pathogen fraction alone. Conditional R² = 0.43, y = 1.11/x^0.25^ – 1, all parameters significant, CI = 95%. Significance of regression was evaluated using a mixed linear model (see ‘Methods’). *n* = 10 for communities, only communities without invasives were used; *n* = 6 for monocultures. Error bars indicate standard errors. Species numberings: 1. *Achillea millefolium*, 2. *Agrostis capillaris*, 3. *Anthoxanthum odoratum*, 4. *Centaurea jacea*, 5. *Holcus lanatus*, 6. *Leucanthemum vulgare*, 7. *Lotus corniculatus*, 8. *Plantago lanceolata*, 9. *Rumex acetosa*, 10. *Trifolium pratense*.

Future experimental PSF research should include community mesocosm experiments. Though results from monocultures can disentangle fundamental processes of PSF, community experiments clearly give very different results (Fig. 4a, b) and seem more representative for field conditions. Especially measurements of PSF in communities of varying biodiversity and functional composition might prove promising. These community experiments are needed to disentangle the mechanisms of how species interactions and community processes influence PSF and subsequently the ERH and invasion success. We propose future experiments should investigate the effect of PSF on invasion of non-native species in communities of differing diversity. Earlier works where a significant effect of PSF on competition of invasive plants was found, often worked with low biodiverse systems^21^ (often one-on-one competition), opposed to our results from a more biodiverse system. We expect that as PSF becomes less negative in more biodiverse communities, most invasive species will benefit less from the ERH and will show lower competitiveness and subsequently lower invasion success. Though labour intensive measurements, it might also prove valuable to include root mass additionally to above-ground biomass in PSF experiments as we have shown here that root mass might be more determining PSF than above-ground or total weight in community.

## Methods

### Monoculture experiment

In order to measure the PSFs that plant species experience when grown alone, a monoculture experiment was set up with 10 native species, typically co-occurring in moist mesotrophic European grasslands (*Achillea millefolium*, *Centaurea jacea*, *Leucanthemum vulgare* (Asteraceae), *Lotus corniculatus*, *Trifolium pratense* (Fabaceae), *Plantago lanceolata* (Plantaginaceae), *Agrostis capillaris*, *Anthoxanthum odoratum*, *Holcus lanatus* (Poaceae), *Rumex acetosa* (Polygonaceae)) from 3 different functional groups (grasses (3 species), leguminous (2) and non-leguminous species (5)). In addition, 3 exotic invasive species in mesotrophic grasslands in Europe (*Avena sterilis* (Poaceae), *Lupinus polyphyllus* (Fabaceae), *Solidago gigantea* (Asteraceae)) were included in the study, one from each of the functional groups mentioned above. Seeds of natives and of *L. polyphyllus* were obtained from Cruydt-Hoeck (The Netherlands), seeds of *A. sterilis* from B&T World Seeds (France), and of *S. gigantea* were collected in the region of Brussels during the autumn of 2021.

The experiment was split into two phases: the conditioning phase, where plants are subjugated to soil microbial communities and when (species-specific) mutualists and pathogens could accumulate, and the response phase, where plants of the same species were grown in the conditioned soil under different soil treatments (Extended Data Fig. 1, see further for the different soil treatments). During the conditioning phase, seeds were germinated on universal potting soil (pH = 6.0, Viano, Belgium) for one to three weeks depending on species, after which they were transplanted into 0.5 l pots (10 × 8 cm, Soparco, France). Plants were grown separately in a sand/inoculum mixture (95-5 vol%) during eight weeks (March and April 2022) in a greenhouse in a randomized setup. The sand fraction did not contain any organic matter, was composed 100% of dried quartz sand, and concentrations of N and P were below detection limits (Type M31, Sibelco NV, Belgium). The inoculum was collected from a moist mesotrophic grassland in a Belgian nature reserve (Doode Bemde; 50.815509, 4.644803). Soil samples (0–10 cm deep) were taken randomly from 10 different locations within this grassland and thoroughly mixed. This soil was stored for 3 days at 5 °C before usage. Plants received a half-strength Hoagland solution^17^ per week, i.e. each plant individual received 2.5 ml per week in the first two weeks, 5 ml per week in the next four weeks and 7.5 ml per week in the last two weeks. At the end of the conditioning phase each individual received a total of 30.0 mg N, 2.0 mg P, 222.0 mg K, 55.3 mg Ca, 31.5 mg Mg, 4.9 mg Fe, 0.03 mg Cu, 0.40 mg B, 0.24 mg Mn, 0.12 mg Zn and 0.07 mg Mo^64^. Pots were watered upon demand two to three times per week into the saucers to avoid leaching out of pathogens and nutrients. At the end of the conditioning phase plants were harvested and disposed. The conditioned soil was used for the response phase.

Three different treatments were applied to the soil: (i) Sterilized soil, by steam sterilization for 1 h at 121 °C in a VAPOUR-line autoclave of VWR. Due to the use of a small soil inoculum in a background of mineral soil, the differences in effect of soil sterilization on nutrient release are negligible^65^. (ii) Untreated soil, where the conditioned soil was directly used to grow plants of the response phase. (iii) pathogen/saprobe filtrate, where soil from the conditioning phase was first filtered through multiple analytical Retsch sieves (mesh sizes of 1000, 500, 250, 180, 125 and 63 µm) with 0.5 l water to retain sand, root particles and other coarse materials. This solution was then filtered through an analytical 20 µm Retsch test sieve—retaining arbuscular mycorrhizal fungi and their spores—after which the filtrate, containing pathogens and non-mycorrhizal microbes that could pass the 20 µm pore-size filter, was collected^17^. After sterilizing the soil (as above), this filtrate was added back. Though nitrogen-fixing bacteria from the genus *Rhizobium* and closely related taxa could also pass through these sieves, visual checks of the roots of all leguminous plants showed the absence of root nodules in all treatments. In this second phase, plants were grown in soil conditioned by the same species during eight weeks, receiving equivalent nutrient dosages as in the first phase, after which they were harvested. The plant material was dried for 72 h at 70 °C prior to weighing above and below-ground dry biomass. For each of the 13 species and three soil treatments, six repetitions were performed for a total of 234 plants in each phase (13 species × 3 soil treatments × 6 repetitions).

### Community experiment

To compare PSF from monocultures with PSF in plant communities, the same 10 native species were grown randomly together in 5 l pots (23 cm diameter, Göttinger, Germany), with one individual per species per pot. The same procedure was followed as for the monoculture experiments unless mentioned otherwise. In the conditioning phase, soil was conditioned by the whole community instead of a single species. Every community received 10 times more nutrient solution as in the monoculture experiment in order to receive the same amount of nutrients per plant. For the response phase, the same three soil treatments as for the monoculture experiment were applied, though an extra factor was included into this design. Some plant communities were assigned an invasive species (one out of three aforementioned invasive species), placed in the middle of the community in a full factorial design for a total of 1200 native plants in each phase (10 species/community × 3 soil treatments × 4 addition possibilities × 10 repetitions) and 90 invasive plants in the second phase (30 plants of each invasive species, 3 soil treatments × 10 repetitions). Invasive plants were included into the plant communities in order to investigate whether they were stronger competitors when PSF was present, and thus if the ERH applies to these invasive species. Above-ground dry biomass was measured for all 1290 individual plants, and below-ground dry biomass for half of all repetitions, thus for 645 plants in total, after careful separation of roots of different species.

### PSF calculations

In this work we distinguish two types of plant-soil feedback: PSF_tot_ is the feedback plants receive from all soil biota, both pathogens and mutualists. It was calculated for each individual plant separately—grown in unsterilized conditioned soil (see above, soil treatment ii)—as the difference between that individuals biomass and the average biomass of the same species when grown in sterilized soil (soil treatment i). Since this difference depends not only on the effect size of feedback, but also on how large a certain plant species becomes, we standardized this term by dividing by the average biomass of the same species when grown in sterilized soil (see Eq. 1). PSF_path_ is the feedback plants receive from only pathogens (and saprobes) and is calculated as PSF_tot_ but with soil treatment ii replaced by treatment iii, i.e. soil treated with the pathogen/saprobe filtrate. Standardized PSF effect sizes were thus measured as follows^65^:

1 $${{St}.{PSF}}_{{species}x}=\frac{\mathop{\sum }\nolimits_{i}^{n}\frac{{{Biomass}}_{{treatment},i}\,-\,{{Biomass}}_{{sterile},{av}}}{{{Biomass}}_{{sterile},{av}}}}{n}$$

All PSFs are calculated using above-ground, below-ground or total biomass, as indicated in captions.

### Statistics

In order to investigate whether PSF measured in monocultures was representative for communities we performed a standardised major axis regression (package smatr^66^) between PSF (PSF_tot_ & PSF_path_) in monocultures and in communities (Fig. 1a, b and Extended Data Fig. 2a, b), after checking for the model assumptions (visual plots and function shapiro.test).

Since PSF in communities was not correlated to PSF in monocultures, we examined whether PSF in community depends on the dominance of the species itself, measured as dry weight biomass. We therefore made mixed linear regressions (function lmer, R package lme4^67^) assessing the relations between PSF and species biomass in communities (Fig. 2a, b and Extended Data Figs. 5, 6, Table 1). Both species biomass (as average dry weight in grams of the sterilized treatment), functional group and their interaction were inserted as fixed factors. As species biomass was inversely transformed (1/x) for improved model fit, a constant of 1 was added to the response variable (standardized effect size of PSF), since the function 1/x cannot exist as a negative. The minimum value for standardized effect size is −1, when an individual would be extremely small when growing in conditioned soil (see Eq. 1). By increasing the standardized effect size in the model by 1 we ensured that the new minimum value of the response variable is 0, the asymptote of the function 1/x. The community to which each individual belonged (as repetition number) and the presence of an invasive species in that community (four levels: no invasive added, *Avena sterilis* added, *Lupinus polyphyllus* added or *Solidago gigantea* added) were inserted as random factors in order to account for differences between communities and the possible impact invasives could have on the reduction in species-specific pathogens. As shown by the negligible amounts of variance explained by these random factors compared to the residual variance in all models and the negligible rise in R² when these variables are added (= difference between conditional R² and marginal R²), these random factors had no significant impact on regressions. Assumptions were visually checked, except for multicollinearity between different fixed values, which was analysed using VIF values with a cut-off of <3. For the relation between the difference in PSF between communities and monocultures and species biomass (Extended Data Fig. 5a, b, Table 1) the same mixed linear regressions were used, but with species biomass (transformed as above) as fixed factor and functional group as random factor.

In order to show that this relation between species biomass and PSF was only present in communities and not in monocultures, we made multiple linear regressions relating PSF and species biomass from the monoculture experiment (Extended Data Fig. 7a, b, Table 1). Explanatory variables were species biomass, functional group and their interaction as above, but without random factors and no transformation of the response variable since this did not improve model fit. Assumptions were checked as for the linear regressions above and with VIF values.

Significant effect sizes of PSF in both monocultures and communities (Extended data Figs. 3, 4) were calculated with one-sample t-tests. PSFs were statistically significant when they were significantly different from 0. Assumptions were checked with the Shapiro-Wilk test. When these were not met, significances were calculated with the Wilcoxon one-sample signed rank test.

The influence of both soil treatment (sterilized, sterilized + pathogen/saprobe filtrate, no treatment) and the presence of an invasive species were analysed using two-way ANOVA’s with interaction term (Fig. 3a, Extended Data Fig. 8a and Table 2) and one-way ANOVA’s (Fig. 3b, Extended Data Fig. 8b and Table 2). An interaction term was added in order to assess whether the effect of invasive species altered depending on the soil treatment. Assumptions were checked as above. Post hoc tests were also performed as above.

Data acquisition was done in Microsoft Excel version 2210 and analyses were performed in R version 4.2.1^68^. All figures were made with ggplot, and edited in Inkscape 1.2.1.

### Limitations of our methods

Pathogen/saprobe-filtrate preparation through a 20 µm pore-size filter is a standardized work method in the field of PSF^8,17,69–72^, but it has its limitations. Sieving a suspension through a 20 µm filter retains micro-arthropods, nematodes, AMF, enchytraeids and collembola, but still contains pathogenic and saprobic soil bacteria and fungi^73–75^. We thus assumed that both saprobic bacteria and fungi had no significant effect on plant growth within these 8 weeks as we assigned the effects observed in PSF_path_ to (species-specific) pathogens. Besides this, some nematode eggs can pass certain smaller pore-sizes^76^, possibly slightly altering the PSF measured, though it has to be verified whether these eggs can have a significant impact on plant growth within 8 weeks. Furthermore, some soil organisms are better at recolonizing the soil after filtration and others are more sensitive to the filtration process^73^, creating a bias in microbe abundance and thus in the PSF measured. Lastly, total native biomass seems lower—though not significantly lower—when grown in the unsterilized soil treatment (= PSF_tot_) than when grown in the sterilized soil treatment with the addition of the pathogen/saprobe filtrate (= PSF_path_) (Fig. 3a, b and Extended Data Fig. 8a, b). This could be attributed to pathogens having to recolonize the soil after addition to the sterilized soil, reducing their negative effect on plant growth. Another, not mutually exclusive, explanation is that some of the filtered pathogens in the soil were more sensitive to the soil processing.

### Supplementary information

Supplementary information

## Data Availability

The data that support the findings of this study are available in figshare with the identifier 10.6084/m9.figshare.23295482.
