# Supplementary material for: Negative plant-soil feedbacks disproportionally affect dominant plants, facilitating coexistence in plant communities
Source: NPJ Biodivers. 2023 Dec 21;2:27. doi: 10.1038/s44185-023-00032-4 (PMC11332034; doi:10.1038/s44185-023-00032-4)
Supplement: Supplementary file 1 — Supplementary information [file 44185_2023_32_MOESM1_ESM.docx]

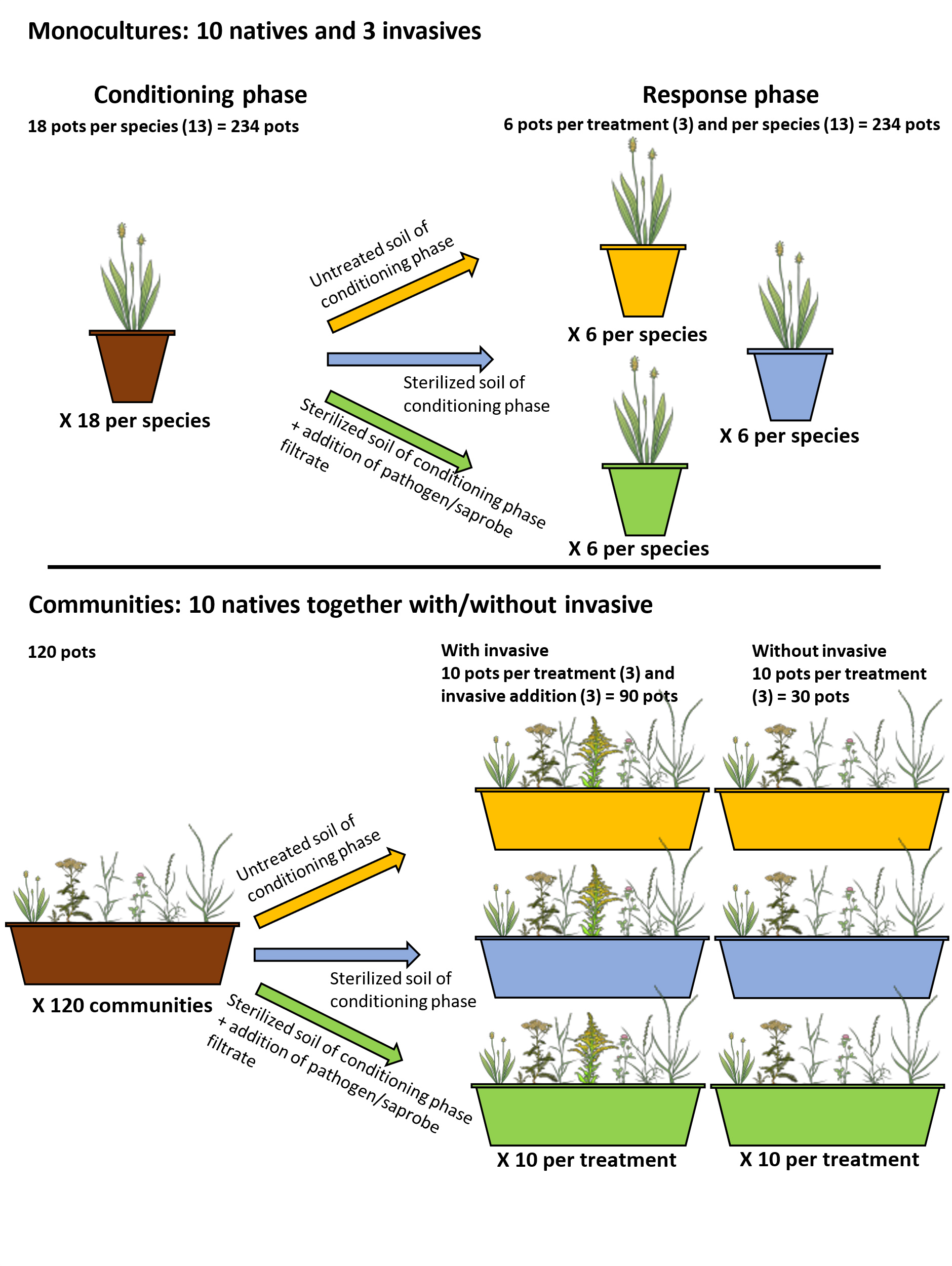


**Extended Data Fig. 1|Setup of the experiment. above**, shows the setup for the monoculture experiment with both phases and soil treatments between both phases. This setup was repeated for each of the 13 species. **below**, shows the setup for the community experiment. Again both phases are shown with all three soil treatments. In the second phase in some pots one invasive species was added (three possible invasive species) and in others no invasive species was added.

**
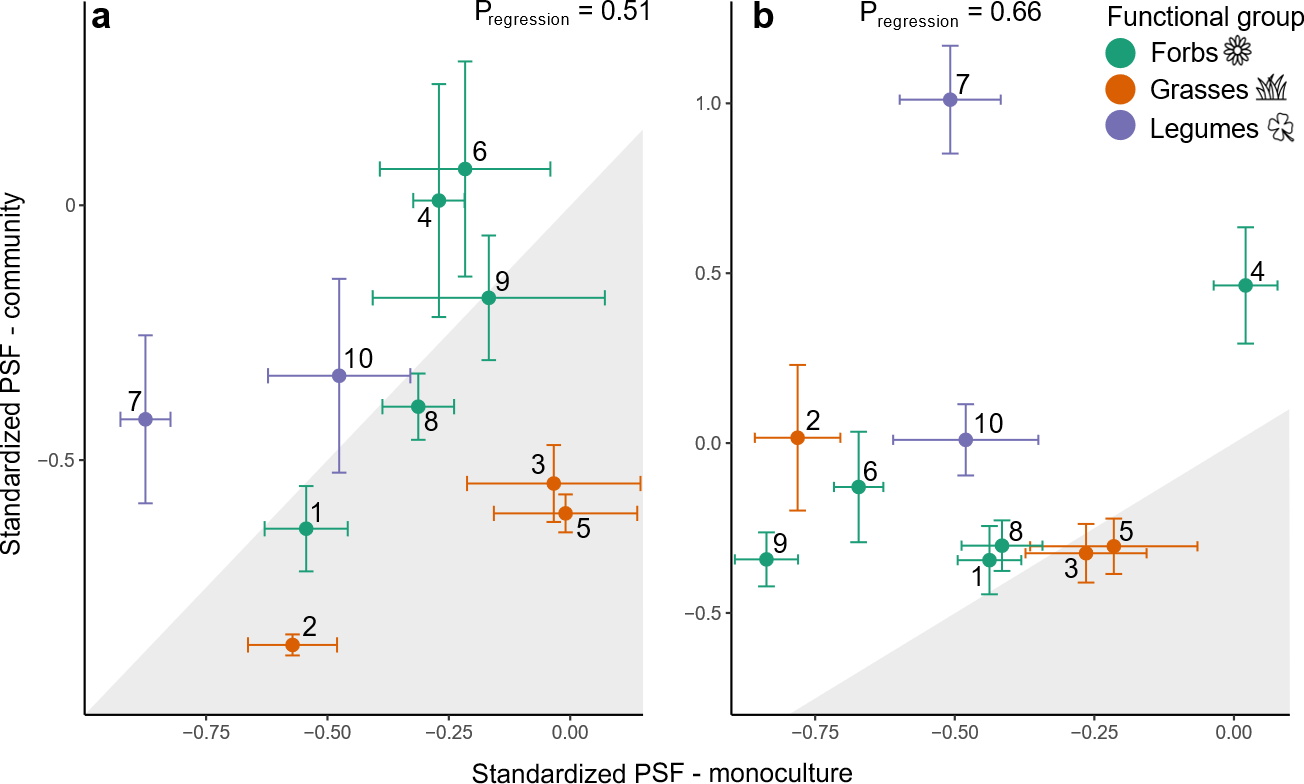
**

**Extended Data Fig. 2|PSFs for all native species grown in community (without invasives) in function of the PSFs of these species when grown alone, calculated for their above ground biomass. a**, shows results for standardized PSF_tot_ from both mutualists and pathogens. **b**, shows results for standardized PSF_path_ from the pathogen fraction alone. Grey areas show when PSF is more positive when grown alone than in community, for white areas PSF is more positive in communities. Significance of correlation was evaluated using a linear model (see Methods). Error bars indicate standard errors, n = 10 for calculation PSF in community per species per soil treatment, only communities without addition of invasives were used; n = 6 for monocultures. Species numberings: 1. *Achillea millefolium*, 2. *Agrostis capillaris*, 3. *Anthoxanthum odoratum*, 4. *Centaurea jacea*, 5. *Holcus lanatus*, 6. *Leucanthemum vulgare*, 7. *Lotus corniculatus*, 8. *Plantago lanceolata*, 9. *Rumex acetosa*, 10. *Trifolium pratense*.


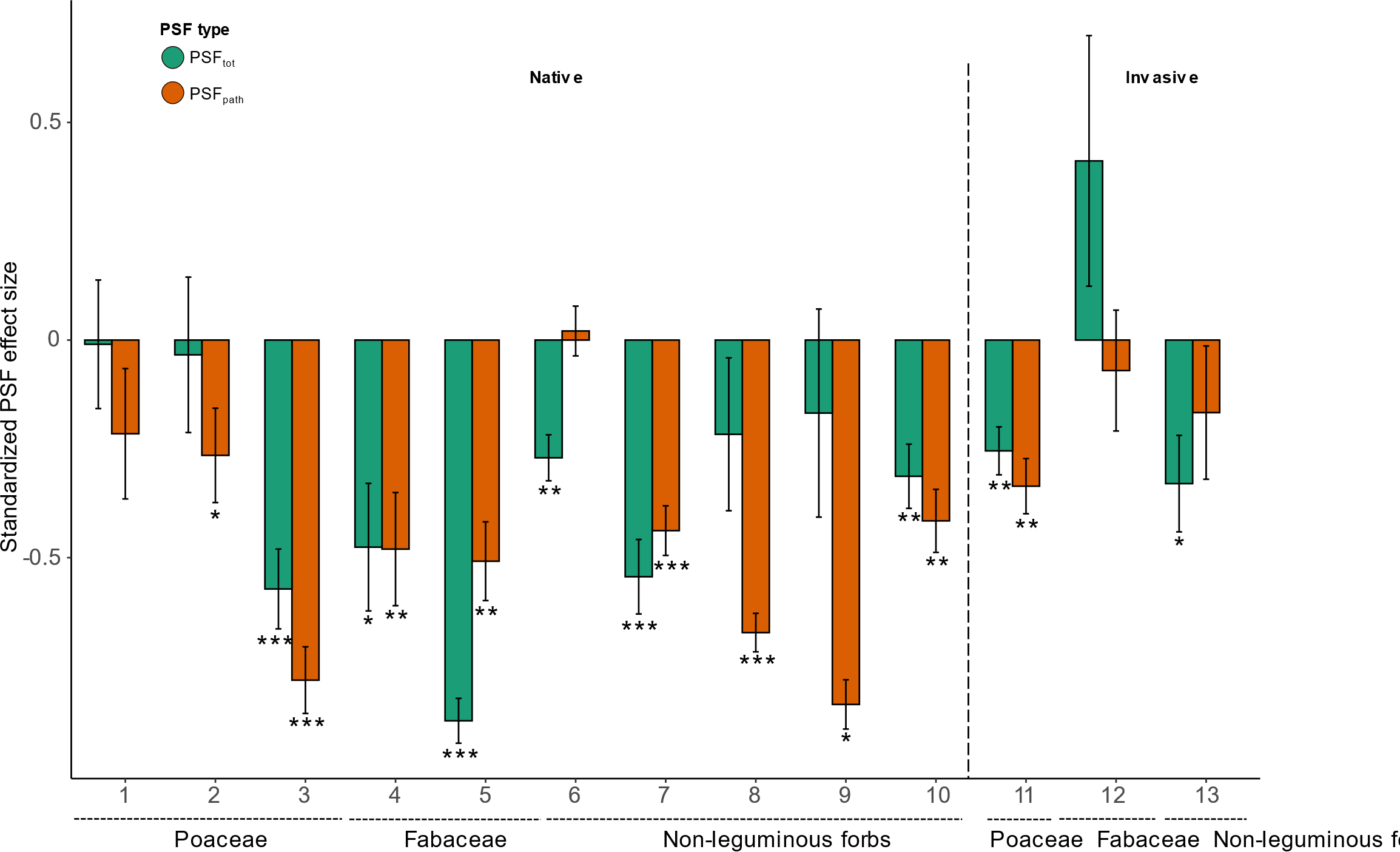


**Extended Data Fig. 3|Standardized PSFs (both PSF_tot_ and PSF_path_) for all species when grown in monoculture, calculated for their above ground biomass.** Standardized PSFs were calculated as in Equation 1, n = 6. Error bars indicate standard error. Statistical significances indicate whether effect size of PSF is significantly different from 0. P-values indication: *** < 0.001 < ** < 0.01 < * < 0.05 < . < 0.1. Numberings: 1. *Holcus lanatus*, 2. *Anthoxanthum odoratum*, 3. *Agrostis capillaris*, 4. *Trifolium pratense*, 5. *Lotus corniculatus*, 6. *Centaurea jacea*, 7. *Achillea millefolium*, 8. *Leucanthemum vulgare*, 9. *Rumex acetosa*, 10. *Plantago lanceolata*, 11. *Avena sterilis*, 12. *Lupinus polyphyllus*, 13. *Solidago gigantea*.


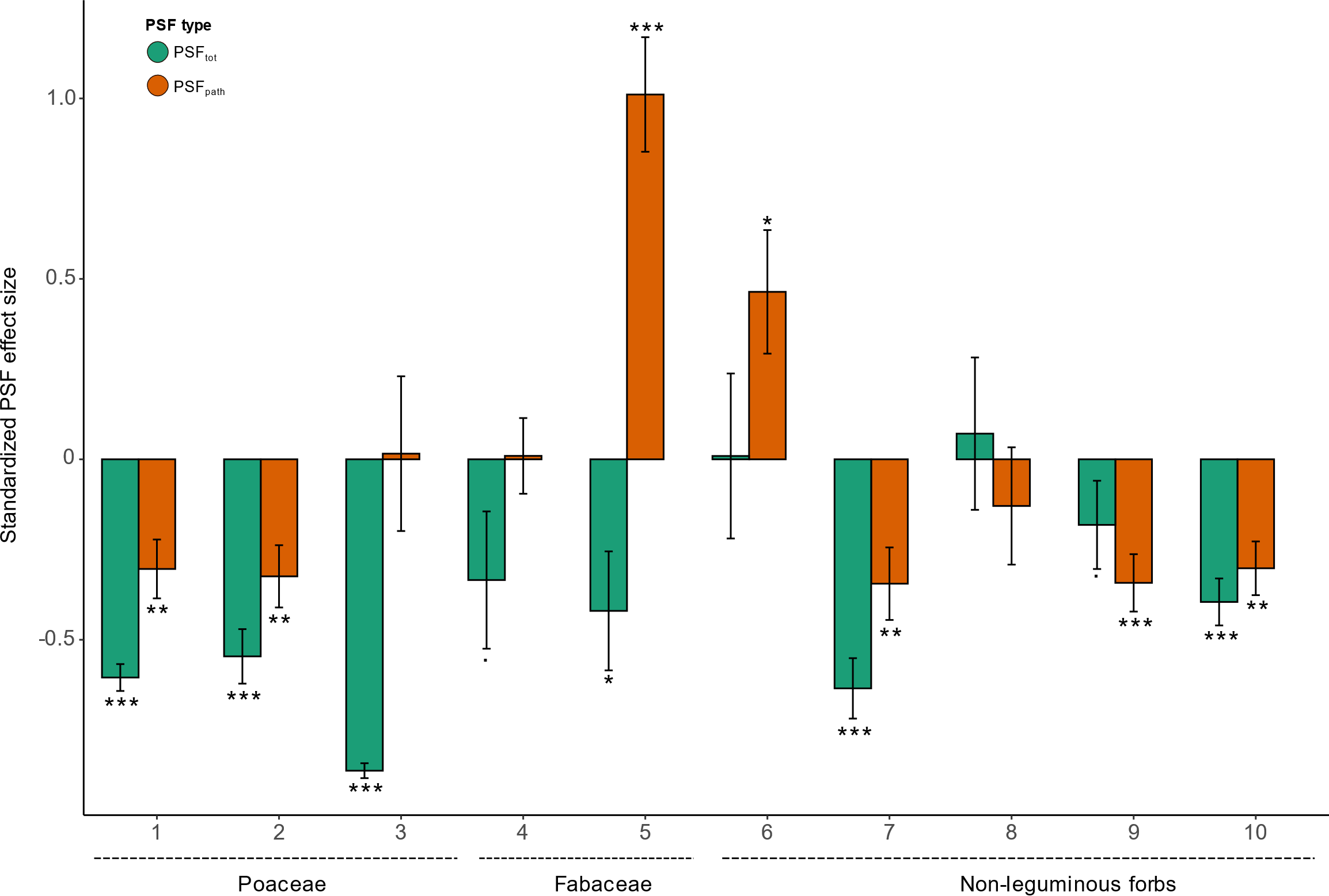


**Extended Data Fig. 4|Standardized PSFs (both PSF_tot_ and PSF_path_) for all native species when grown in community without invasive species, calculated for their above ground biomass.** Standardized PSFs were calculated as in Equation 1, n = 10. Error bars indicate standard error. Statistical significances indicate whether effect size of PSF is significantly different from 0. P-values indication: *** < 0.001 < ** < 0.01 < * < 0.05 < . < 0.1. Numberings: 1. *Holcus lanatus*, 2. *Anthoxanthum odoratum*, 3. *Agrostis capillaris*, 4. *Trifolium pratense*, 5. *Lotus corniculatus*, 6. *Centaurea jacea*, 7. *Achillea millefolium*, 8. *Leucanthemum vulgare*, 9. *Rumex acetosa*, 10. *Plantago lanceolata*.


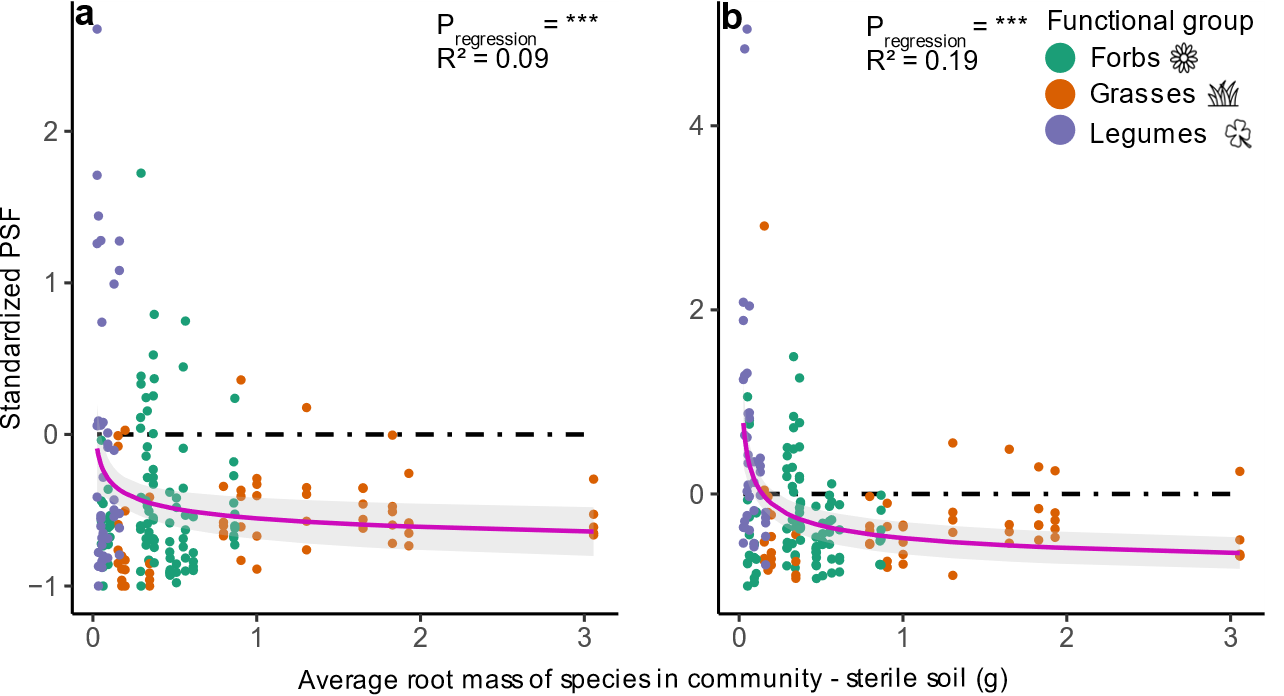


**Extended Data Fig. 5|PSFs for all native individuals grown in community in function of their averaged biomass when grown in sterile soil, calculated for their below ground biomass. a**, shows results for standardized PSF_tot_ from both mutualists and pathogens. R² indicated on the graph is the marginal R², conditional R² = 0.10. Trendline was made with the function nls, y = 0.45/x^0.19^ – 1, all parameters highly significant, CI = 99.5%. **b**, shows results for standardized PSF_path_ from the pathogen fraction alone. Conditional R² = 0.19, y = 0.52/x^0.33^ – 1, all parameters highly significant, CI = 99.5%. Significance of correlation was evaluated using a mixed linear model (see Methods).


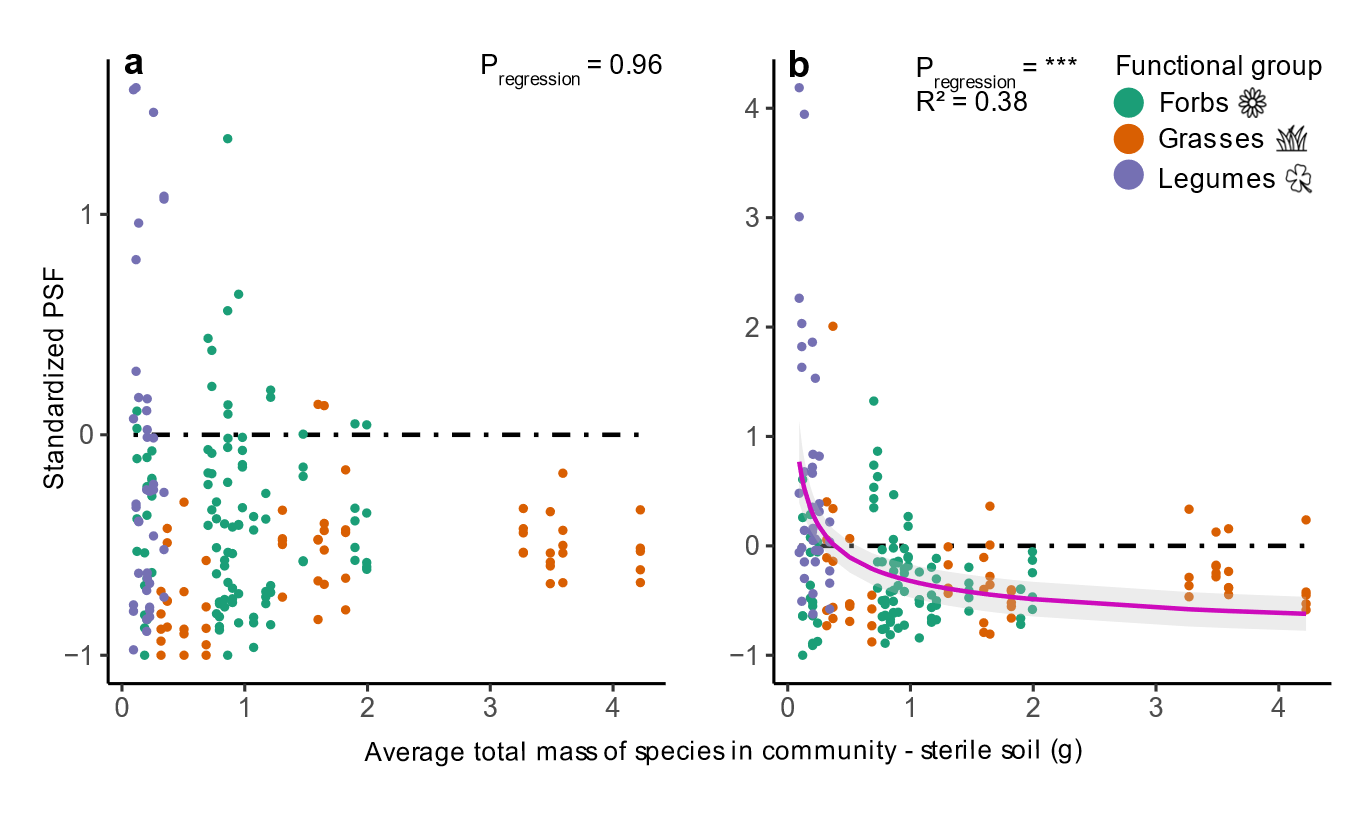


**Extended Data Fig. 6|PSFs for all native individuals grown in community in function of their averaged biomass when grown in sterile soil, calculated for their total biomass. a**, shows results for standardized PSF_tot_ from both mutualists and pathogens. **b**, shows results for standardized PSF_path_ from the pathogen fraction alone. Significance of correlation was evaluated using a mixed linear model (see Methods). R² indicated on the graph is the marginal R², conditional R² = 0.40. Trendline was made with the function nls, y = 0.68/x^0.40^ – 1, all parameters highly significant, CI = 99.5%.


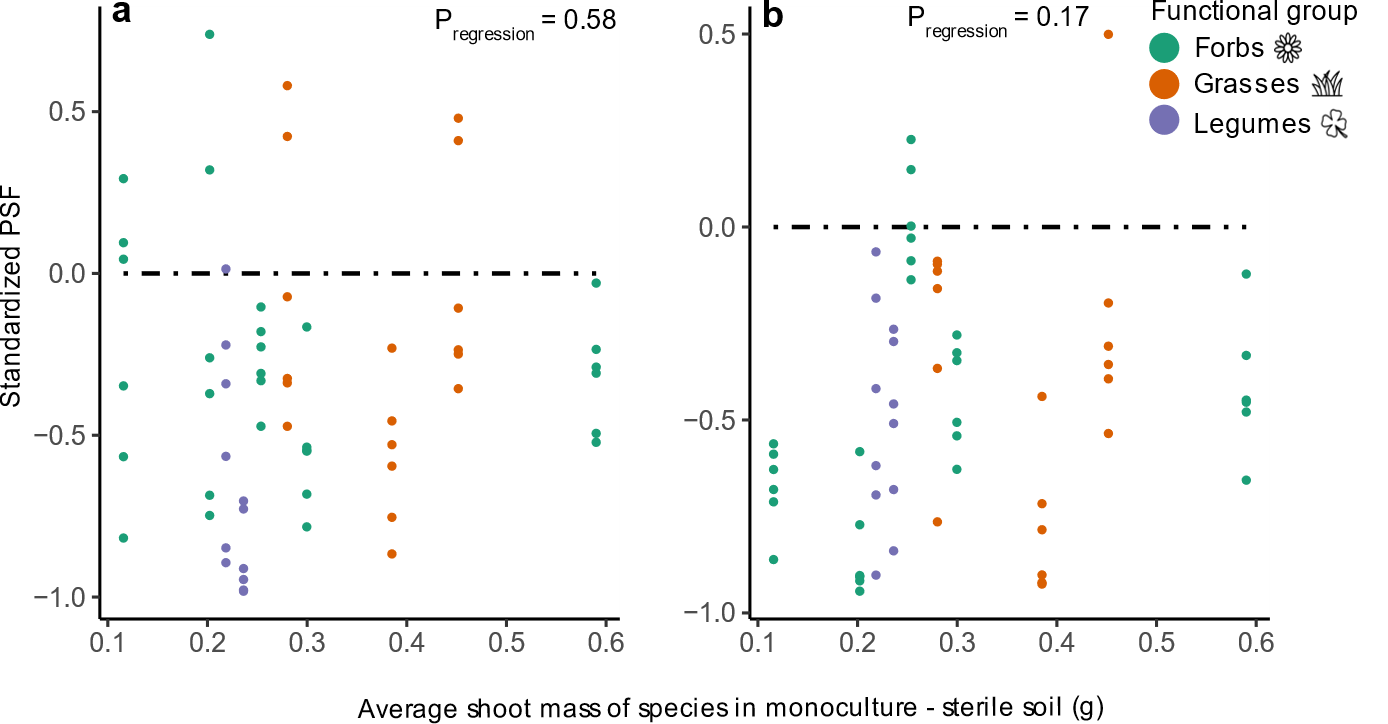


**Extended Data Fig. 7|PSFs for all native individuals grown alone in function of their averaged biomass when grown in sterile soil, calculated for their above ground biomass.** **a**, shows results for standardized PSF_tot_ from both mutualists and pathogens. **b**, shows results for standardized PSF_path_ from the pathogen fraction alone. Significance of correlation was evaluated using a multiple linear model (see Methods).


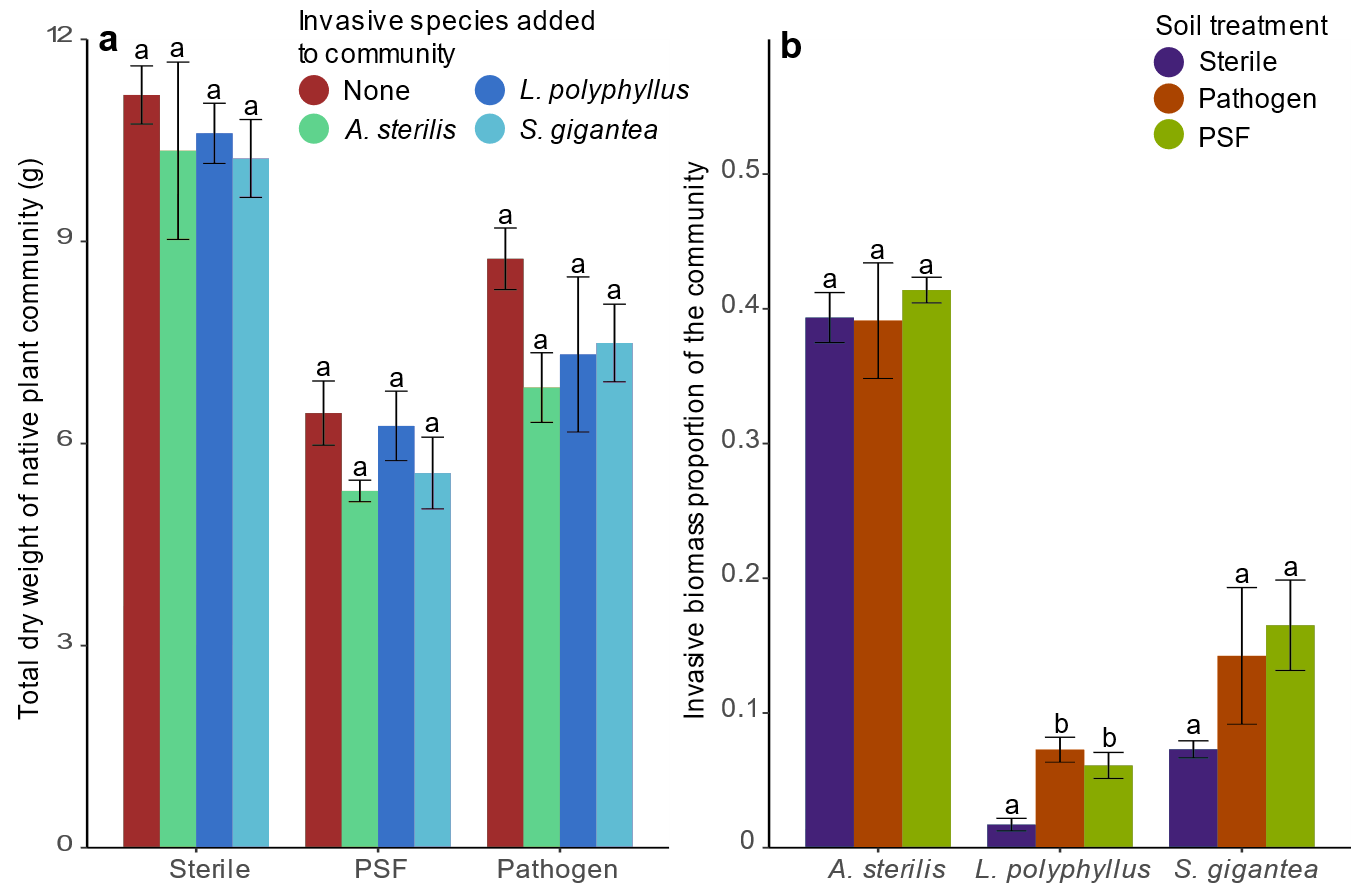


**Extended Data Fig. 8|Impact of invasives and different soil treatments on natives, calculated for total biomass. a**, biomass of all natives combined in function of both soil treatment and presence/absence of an invasive. Sterile = sterilized soil treatment, PSF = unsterilized soil treatment, Pathogen = sterilized soil treatment + addition of pathogen/saprobe filtrate. **b**, proportion of invasives (%) in function of soil treatment. Letters indicate significant differences depending on (a) the presence of an invasive species or (b) soil treatment, calculated with Tukey’s HSD test, α = 0.05, n = 5. Error bars indicate standard error. *A. sterilis* = *Avena sterilis*, *L. polyphyllus* = *Lupinus polyphyllus*, *S. gigantea* = *Solidago gigantea*.
